# Microencapsulation of Erythrocytes Extracted from *Cavia porcellus* Blood in Matrices of Tara Gum and Native Potato Starch

**DOI:** 10.3390/foods11142107

**Published:** 2022-07-15

**Authors:** Carlos A. Ligarda-Samanez, Elibet Moscoso-Moscoso, David Choque-Quispe, Henry Palomino-Rincón, Edgar L. Martínez-Huamán, Mary L. Huamán-Carrión, Diego E. Peralta-Guevara, Jimmy Aroni-Huamán, José C. Arévalo-Quijano, Wilbert Palomino-Rincón, Germán De la Cruz, Betsy S. Ramos-Pacheco, Jenny C. Muñoz-Saenz, Mauricio Muñoz-Melgarejo

**Affiliations:** 1 Food Nanotechnology Research Laboratory, Universidad Nacional José María Arguedas, Andahuaylas 03701, Peru; elibetmm22@gmail.com (E.M.-M.); mhuaman@unajma.edu.pe (M.L.H.-C.); 2 Agroindustrial Engineering, Universidad Nacional José María Arguedas, Andahuaylas 03701, Peru; dchoque@unajma.edu.pe (D.C.-Q.); hpalomino@unajma.edu.pe (H.P.-R.); jaroni@unajma.edu.pe (J.A.-H.); bsramos@unajma.edu.pe (B.S.R.-P.); 3 Water Analysis and Control Research Laboratory, Universidad Nacional José María Arguedas, Andahuaylas 03701, Peru; diepltagvra@gmail.com; 4 Department of Education and Humanities, Universidad Nacional José María Arguedas, Andahuaylas 03701, Peru; emartinez@unajma.edu.pe (E.L.M.-H.); jcarevalo@unajma.edu.pe (J.C.A.-Q.); 5 Agricultural and Livestock Engineering, Universidad Nacional San Antonio Abad, Cusco 08000, Peru; wilbert.palomino@unsaac.edu.pe; 6 Agricultural Science Facultad, Universidad Nacional San Cristobal de Huamanga, Ayacucho 05000, Peru; german.delacruz@unsch.edu.pe; 7 Department of Human Medicine, Universidad Peruana los Andes, Huancayo 12006, Peru; d.jmunoz@upla.edu.pe (J.C.M.-S.); d.mmunoz@upla.edu.pe (M.M.-M.)

**Keywords:** encapsulation, *Cavia porcellus*, erythrocytes, iron, spray drying

## Abstract

Ferropenic anemy is the leading iron deficiency disease in the world. The aim was to encapsulate erythrocytes extracted from the blood of *Cavia porcellus*, in matrices of tara gum and native potato starch. For microencapsulation, solutions were prepared with 20% erythrocytes; and encapsulants at 5, 10, and 20%. The mixtures were spray-dried at 120 and 140 °C. The iron content in the erythrocytes was 3.30 mg/g and between 2.32 and 2.05 mg/g for the encapsulates (*p* < 0.05). The yield of the treatments varied between 47.84 and 58.73%. The moisture, water activity, and bulk density were influenced by the temperature and proportion of encapsulants. The total organic carbon in the atomized samples was around 14%. The particles had diverse reddish tonalities, which were heterogeneous in their form and size; openings on their surface were also observed by SEM. The particle size was at the nanometer level, and the zeta potential (ζ) indicated a tendency to agglomerate and precipitation the solutions. The presence of iron was observed on the surface of the atomized by SEM-EDX, and FTIR confirmed the encapsulation due to the presence of the chemical groups OH, C-O, C-H, and N-H in the atomized. On the other hand, high percentages of iron release in vitro were obtained between 88.45 and 94.71%. The treatment with the lowest proportion of encapsulants performed at 140 °C obtained the best results and could potentially be used to fortify different functional foods.

## 1. Introduction

Iron is a fundamental micronutrient for humans, as it is involved in a wide variety of metabolic processes, including oxygen transport, deoxyribonucleic acid (DNA) synthesis, and electron transport [1]. It promotes the production of proteins, hemoglobin, and oxygen transport, regulates cell development [2], neuronal [3], and helps eliminate carbon dioxide. Insufficiency [4] or overexposure to iron [5] has notorious effects on human health [6] since it causes damage to the heart, liver, and central nervous system. This condition is determined by absorption, metabolism, and degree of interaction in physiological processes [7].

Dietary iron comes in two forms: heme and non-heme [8]; the main sources of heme iron are hemoglobin and myoglobin resulting from the consumption of red meat, poultry, and fish, while non-heme iron is obtained from cereals, legumes, fruits, and vegetables. Heme iron is highly bioavailable with a rate between 15–35%, and dietary factors have little effect on its assimilation, while the absorption [9] of non-heme iron is much lower with the uptake of 2–20%, strongly influenced by the presence of other food components such as ascorbic acid [10]. The amount of non-heme iron in a daily diet is higher than the amount of heme iron. Thus, despite its lower bioavailability [11], non-heme iron generally contributes more to nutrition. The main inhibitors of iron absorption are phytic acid, polyphenols, calcium, zinc, and partially digested protein peptides [12]. On the other hand, absorption enhancers are ascorbic acid and muscle tissue [13] that can reduce ferric iron to ferrous iron and bind it into soluble complexes available for absorption [14].

Food fortification and oral iron supplementation are the most used mechanisms for the prevention and treatment of anemia, and they are performed to protect and release the mineral at the right time and place [15], with encapsulation being the most used method, which allows inhibiting the different mechanisms of reaction and degradation of bioactive compounds, during the incorporation of ingredients to food systems, this process allows protecting the nucleus with various matrices such as starches, maltodextrins, gums, lipids, carbohydrates, proteins and antioxidants [16]. The most commonly applied encapsulation methods in the food industry are emulsification [17], gelation [18], extrusion [19], spray drying [20] and lyophilization [21], and there are also chemical encapsulation techniques such as coacervation, co-crystallization, interfacial polymerization, ionic gelation, polymeric incompatibility, liposome entrapment and molecular inclusion [22].

Guinea pigs (*Cavia porcellus*) have nutritional importance in the diet of Andean populations; they are a source of iron, proteins, and other nutrients. They also contribute to the economic income of the families that are dedicated to their breeding, although their consumption is not yet widespread throughout the world [23,24,25], Tara gum is a galactomannan, which is a neutral reserve polysaccharide extracted from the endosperm of the seed of the *Caesalpinia spinosa* tree; it is characterized by possessing the main chain of β-D-mannose units with glycosidic bonds [26], Native potato starch (*Solanum tuberosum* spp. *Andígena*) is a derivative used at industrial and domestic levels due to its gelatinization properties and low tendency to retrograde [27].

**Figure 1:**
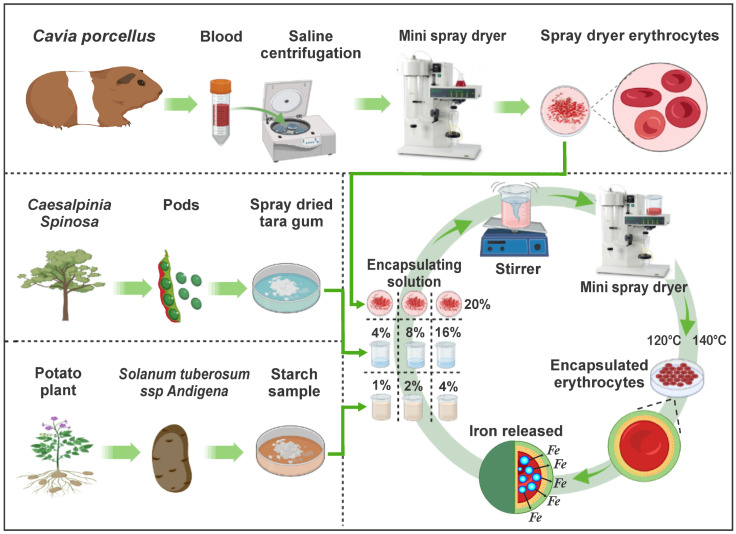
Experimental flow diagram.

In recent years, the development of technologies to preserve and enrich nutrients through encapsulation has increased, recommending combinations of heme and non-heme iron [28,29]. That is why this research aimed to extract iron-rich erythrocytes from *C. porcellus* blood, then encapsulate them in matrices of tara gum and native potato starch, which would give value to the raw materials, potentially using them in the fortification of various products. The complete experimental flow chart is shown in Figure 1.

## 2. Materials and Methods

### 2.1. Materials

The guinea pig (*Cavia porcellus*) blood from the Peru line was kindly provided by the Municipal Camal of the district of San Jeronimo, which is authorized by the national agricultural safety service of Peru (SENASA) and was collected under totally safe and aseptic conditions. The tara (*Caesalpinia spinosa*) was kindly provided by local farmers of the district of Ocobamba. The native potato (*Solanum tuberosum* spp. *Andígena*) of the yanapalta variety, was acquired in the central market of the district of Andahuaylas and was suitable for consumption; All the districts above belong to the Apurimac region of Peru. The other chemical inputs used in the trials were of analytical grade. The ethics committee of the Universidad Nacional José María Arguedas de Andahuaylas, Peru, approved the use of animals in this research by Resolution No. 232-2020-CO-UNAJMA on 22 September 2020.

### 2.2. Obtaining Spray-Dried Erythrocytes

**Figure 2:**
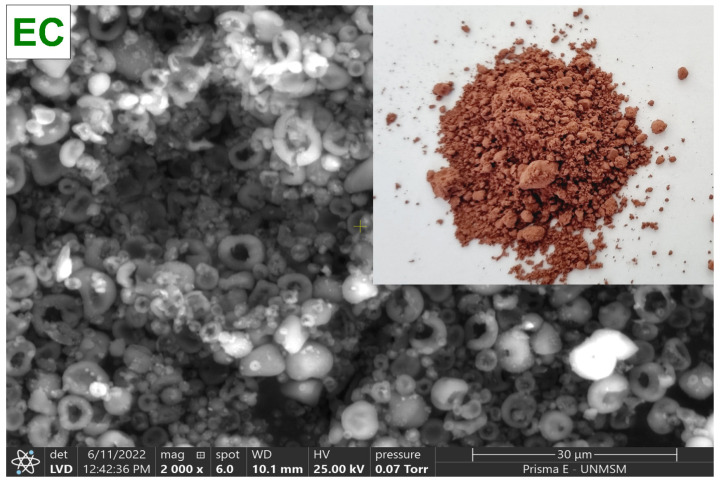
Erythrocytes of *Cavia porcellus* spray-dried (EC).

To collect the *C. porcellus* blood, sodium citrate was used as an anticoagulant agent (3 g/L). The globular package was separated by centrifugation at 3000 rpm for 10 min in a CR4000R centrifuge (Centurion, Pocklington, UK), and then it was washed with NaCl at 0.9%. The centrifuged blood was then diluted in a saline solution, and the viscosity was adjusted to 30 cps with a DV-E rotational viscometer (Brookfield Engineering Laboratories, Inc., Stoughton, MA, USA) with spindle N° 61, for subsequent drying in a B-290 mini spray dryer (BÜCHI Labortechnik AG, Flawil, Switzerland), at an inlet temperature of 120 °C, 100% suction rate, 30% pumping rate, and a 0.7 mm diameter nozzle. Finally, the atomized material was collected in low-density polyethylene bags and stored in a desiccator at 20 °C until further use. Figure 2 shows the spray-dried *C. porcellus* erythrocytes (EC).

### 2.3. Obtaining Spray-Dried Tara Gum

A total of 30 g of tara seeds was added without germ in 800 mL of distilled water to mix them with an M6 thermo-magnetic stirrer (CAT, Ballrechten-Dottingen, Germany) for 12 h at 80 °C until a gum was obtained. Subsequently, the extract was filtered and purified, mixed with 96% ethanol in a 1:1 ratio. This procedure was performed in order to precipitate the gum, which was diluted in water and adjusted to a viscosity of 30 cps with a DV-E rotational viscometer (Brookfield Engineering Laboratories, Inc., Stoughton, MA, USA) with spindle N° 61. Then, it was dried in a B-290 mini spray dryer (BÜCHI Labortechnik AG, Flawil, Switzerland) at an inlet temperature of 100 °C, suction rate of 100%, the pumping rate of 15%, and a 0.7 mm diameter nozzle. Finally, the atomized material was collected in low-density polyethylene bags and stored in a desiccator at 20 °C until further use.

### 2.4. Obtaining Starch from Native Potatoes

5 kg of native potatoes of the yanapalta variety were weighed and washed, then peeled and cut into small pieces in order to homogenize them in a blender silentmixx (Bosch, Stuttgart, Germany). Subsequently, successive washes were made with distilled water to separate the starch by sedimentation, which was dried at 50 °C in a forced convection oven FED 115 (BINDER, Tuttlingen, Germany), and ground to a fine powder, which was sieved through 45 µm mesh in an analytical sieve shaker AS 200 (Retsch, Haan, Germany). Finally, the starch was collected in low-density polyethylene bags and stored in a desiccator at 20 °C until further use.

### 2.5. Erythrocytes Encapsulation

The spray-dried encapsulates were obtained considering a constant amount of erythrocytes (core material), varying the amount of the encapsulants tara gum and native potato starch, in the proportions shown in Table 1, preparing solutions of different concentrations (25, 30, and 40% *w/v*), to be dried at inlet temperatures of 120 and 140 °C, respectively.

For the encapsulation of erythrocytes, solutions were prepared with native potato starch and tara gum at different concentrations (5, 10, and 20% *w/v*). The erythrocytes were dispersed in the encapsulating solution until a homogeneous mixture was obtained. Then, the viscosity was adjusted to 30 cps with a DV-E rotational viscometer (Brookfield Engineering Laboratories, Inc., Stoughton, MA, USA) with spindle N° 61, It was then dried in a B-290 mini spray dryer (BÜCHI Labortechnik AG, Flawil, Switzerland) at inlet temperatures of 120 and 140 °C, 100% suction rate, 30% pumping rate, and a 0.7 mm diameter nozzle.

Finally, the encapsulated material was collected in low-density polyethylene bags and stored in a desiccator at 20 °C until further use. The microencapsulates obtained were named T1C, T2C, T3C, T4C, T5C, and T6C.

### 2.6. Iron Content

200 mg of sample was weighed in a beaker, then 3 mL of HCl and 9 mL of HNO_3_ were added, completed with ultrapure water up to a volume of 50 mL, which was then digested in a microwave digester (SCP Science, Miniwave, QC, Canada). Subsequently, iron was read in an inductively coupled plasma optical emission spectrophotometer ICP-OES 9820 138 (Shimadzu, Kyoto, Japan). Previously, a calibration curve was developed from 0 to 50 mg/L with a 2% iron standard solution in HNO_3_. Finally, the analysis was performed in axial mode, with a sample exposure of 30 s, argon gas flow of 10 L/min, and a wavelength of 239.562 nm. The iron content was calculated with the following formula:

(1) $$\mathrm{Ct}=C\cdot \left(\frac{V}{m}\right),$$

where: Ct, total iron content (mg/g); C, iron content (mg/L); V, volume used for digestion (L), and m, sample mass (g).

### 2.7. Moisture, Water Activity (Aw) and Bulk Density

Moisture content was determined by the official AOAC 950.10 method [30], by the difference in weight of the initial and final sample, dried in a forced convection oven FED 115 (BINDER, Tuttlingen, Germany) at 105 °C. Water activity was determined using the HygroPalm23-AW water activity meter (Rotronic brand, Bassersdorf, Switzerland). Bulk density was determined by placing a known amount of encapsulates in a 10 mL graduated cylinder, then repeatedly tapping on a flat surface, recording the mass in grams and the volume.

### 2.8. Solubility

It was determined by solubilizing 2.5 g of encapsulates in 250 mL of distilled water, then stirred for 5 min until a homogeneous solution was obtained; subsequently, the supernatant was separated by centrifugation at 5000 rpm for 5 min in a CR4000R centrifuge (Centurion, Pocklington, UK). Finally, an aliquot of the supernatant was dried in a forced convection oven FED 115 (BINDER, Tuttlingen, Germany) at 105 °C for 5 h. The following equation was used for the calculation:

(2) $$S=\left(\frac{m_{2}}{m_{1}}\right)\cdot 100,$$

where: S is the solubility percentage (%), m_1_ is the initial weight of the encapsulated powder, and m_2_ is the weight of the dry powder after dissolution.

### 2.9. Hygroscopicity

In a hermetic container, 1 g of sample was dispersed in 100 mL of a saturated NaCl solution (75% relative humidity). It maintained the temperature at 25 °C. After 7 days, the samples were weighed again and expressed as a percentage increase in mass.

(3) $$I=\left(\frac{m_{3-}m_{2}}{m_{2}-{\;m}_{1}}\right)\cdot 100,$$

where: I is the mass increment (%), m_1_ is the weight of the empty Petri dish, m_2_ is the weight of the Petri dish + sample, and m_3_ is the weight of the Petri dish + sample after seven days.

### 2.10. Yield of Encapsulation

It was calculated, considering the weight of the final atomized powder concerning the initial total weight, according to the following equation:

(4) $$Y\;\left(\%\right)=\left(\frac{\mathrm{Pi}}{\mathrm{Pf}}\right)\cdot 100,$$

where: Y, percentage yield (%); Pi, initial weight of materials (g) and Pf, the weight of final atomized powder (g).

### 2.11. Color

The color profile parameters of erythrocytes and encapsulates were determined on the CIE L*a*b color scale and measured with a CR-5 colorimeter (Konica Minolta, Tokyo, Japan). Considering: L* lightness (0 = black and 100 = white), a* chroma (+a = red, −a = green) and b* chroma (+b = yellow and −b = blue).

### 2.12. Total Organic Carbon

The samples studied were previously homogenized, then 50 mg of each material were weighed in ceramic containers, to be later analyzed in a total organic carbon analyzer TOC-L CSN-SSM 5000A (Shimadzu, Kyoto, Japan), with a flow of oxygen of 150 mL/min through the software TOC control L V. 1.07.

### 2.13. SEM-EDX Analysis

The morphology of the erythrocytes and encapsulates was analyzed using a Prism E scanning electron microscope (SEM) (Thermo Fisher, Massachusetts, MA, USA) at an accelerating voltage of 25 kV and a magnification of 1000×. The energy-dispersive X-ray spectroscopy (EDX) module of the equipment above was used for the chemical analysis of the particle surface.

### 2.14. Particle Size and ζ Potential

Particle size and ζ potential measurements of the spray-dried encapsulates were determined using a nano ZLS Z3000 dynamic light scattering (DLS) instrument (Nicomp, Danvers, MA, USA).

### 2.15. FTIR Analysis

The functional groups of tara gum, native potato starch, erythrocytes, and encapsulates were analyzed with a Fourier transform spectrophotometer (FTIR), Nicolet IS50 (ThermoFisher, Waltham, MA, USA), using the transmission modulus in the range of 400 to 4000 cm^−1^, with a resolution of 8 cm^−1^, 32 scans and using 0.1% KBr tablets.

### 2.16. Thermal Analysis

It was taken 10 mg of encapsulates and erythrocytes in order to analyze the thermal stability of the materials by TGA (thermogravimetric analysis) and DTA (differential thermal analysis), in a temperature range between 20 and 600 °C, in an N_2_ atmosphere, with a heating rate of 10 °C/min, using a TGA 550 thermal analyzer (TA Instrument, New Castle, DE, USA).

### 2.17. In Vitro Bioavailability

For estimation of bioavailability in vitro, 50 mg of microencapsulates obtained immediately after spray drying was dispersed in 500 mL of 0.1 N HCl and stirred until completely homogeneous. Subsequently, the samples were placed in a water bath with a WTB 50 stirring system (Memmert, Schwabach, Germany). Samples were extracted at 0, 30, 60, 60, 90, and 120 min for afterward reading in an inductively coupled plasma optical emission spectrophotometer ICP-OES 9820 138 (Shimadzu, Tokyo, Japan). The analysis was performed in axial mode, with a sample exposure of 30 s and an argon gas flow of 10 L/min at a wavelength of 239.562 nm. The results were expressed as the percentage of initial iron dissolved in 0.1 N HCl, which was calculated with the following formula:

(5) $$L=\left(\frac{C_{T}}{C_{0}}\right)\cdot 100,$$

where: L, iron content in time (%); $C_{T}$, iron content in time (mg/g) and, $C_{0},$ initial iron content (mg/g).

### 2.18. Statistical Analysis

Origin Pro 2022 software (OriginLab Corporation, Northampton, MA, USA) was used, and analysis of variance (ANOVA) and Tukey’s multiple range test at 5% significance (*p* < 0.05) were used; all results were obtained in triplicate.

## 3. Results and Discussion

### 3.1. Iron Content

**Figure 3:**
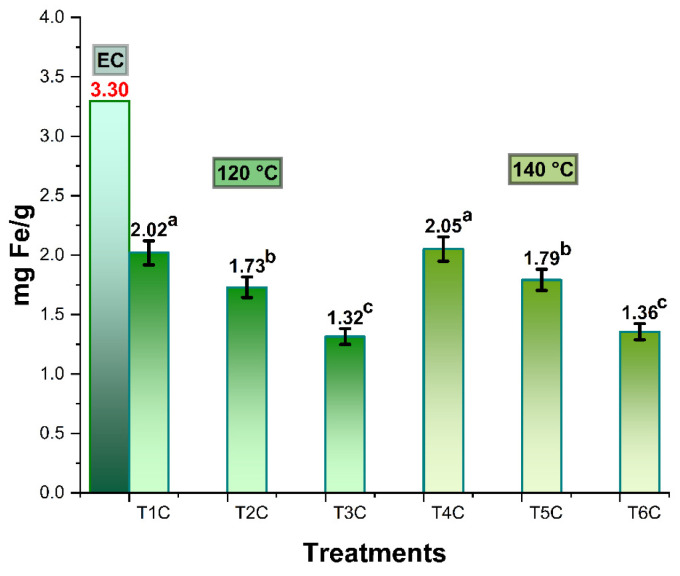
Iron content in spray-dried erythrocytes (EC), encapsulated a 120 °C (T1C, T2C y T3C) and 140 °C (T4C, T5C y T6C). Different letters indicate a significant difference.

Figure 3 shows the iron content in spray-dried *Cavia porcellus* blood erythrocytes, which was 3.30 mg/g, higher than the 2.49 mg/g reported for commercial bovine erythrocytes [28]. In the case of the encapsulation treatments at inlet temperatures of 120 and 140 °C, it was between 1.32–2.02 mg/g and 1.36–2.05 mg/g, respectively; significant differences were also observed in all the treatments of each temperature block (*p* < 0.05). This behavior would be due to the proportions of the matrices used [31], noting that the lower the amount of encapsulant, the higher the iron content in the encapsulates [28] since spray drying at high temperatures would allow a higher concentration of iron content, due to the greater loss of moisture during drying.

Spray-dried erythrocytes are considered a safe and adequate source of heme iron (up to 99%), which is why it is recommended to obtain it from different animal sources, and its subsequent encapsulation through methods that improve the release and absorption of this important nutrient [29,32,33,34]. The results obtained in the present investigation were superior to those reported for bovine erythrocytes (EB) encapsulated in maltodextrin, whose maximum value for 20% EB was 0.77 mg Fe/g [28]; on the other hand, much higher iron contents were reported in the literature for encapsulated ferrous sulfate heptahydrate and ferrous fumarate in various polymeric matrices [28,31,35,36,37]. Nevertheless, it is recommended that a combination of erythrocytes from animal blood and non-heme iron sources be used to achieve greater stability and bioavailability [28,38]. Iron absorption occurs mainly in the duodenum, and it is known that heme iron enhances the absorption of non-heme iron, the latter being much less consumed in developing countries [39]. A daily intake of 10 to 15 mg of iron in the human diet is recommended, so the erythrocytes obtained in the present research could be used in fortified foods with prior food safety studies [15,40,41]. Microencapsulation is one of the most recommended techniques to achieve a controlled release of iron [42,43,44,45].

### 3.2. Moisture, Water Activity (Aw) and Bulk Density

Table 2 shows the results of moisture, Aw, and bulk density, in which significant differences were observed due to the influence of the inlet temperature (*p* < 0. 05), regarding these three physical properties, they are considered essential during the storage of dry products, so it is recommended that moisture be below 5% [46,47,48]. On the other hand, Aw is related to biological and chemical deterioration mechanisms, such as enzymatic and non-enzymatic browning, high values would indicate the greater availability of free water, capable of participating in different reaction mechanisms, it is considered that in atomized products this value be lower than 0.6 [49,50]. The water content in the erythrocytes was 11.30% and in the encapsulates it varied between 4.73 and 8.61%, the Aw for all the samples was between 0.36 and 0.43, observing that with the increase of temperature and proportion of encapsulates the Aw decreases [51,52]. The values for bulk density ranged between 0.17 and 0.24, showing that the treatments with smaller particle sizes presented higher [48,53].

### 3.3. Solubility and Hygroscopicity

Solubility is an essential property in food products. Table 3 shows results between 43.22 and 57.18% for the encapsulates, Solubility results increased with increasing temperature because of the spray drying conditions [43,54,55], the solubility rates were below the value reported for ferrous sulfate encapsulated in maltodextrin and potato starch, which was around 95% [31]. As far as hygroscopicity was concerned, values between 22.05 and 32.94% were reported, which are slightly above the critical value of 20%, which is the desired value during storage and handling of dehydrated products [20,31].

### 3.4. Yield of Encapsulation and Color

Table 4 shows the yields of the encapsulates, which were between 47.84 and 58.73%; the latter result corresponding to treatment T4C was obtained at 140 °C. Lower results (between 39 and 47%) were reported for bovine erythrocytes encapsulated in maltodextrin [28]. On the other hand, very similar results, between 47.93 and 56.26%, were reported for ferrous sulfate encapsulated in potato starch and maltodextrin [31].

As far as color was concerned, can also be seen t significant differences in the encapsulates, observing that the lower the amount of encapsulants, the darker the reddish color of the atomized powders, which is typical of the use of erythrocytes from animal blood [28,38]. The study of color is relevant to the organoleptic properties of products fortified with iron encapsulants [28,31]. In addition, they are affected by the natural color of the wall materials used [16,56,57], and the amount of these used in spray drying [58,59], which would cause changes in the color shades due to non-enzymatic browning reactions at high temperatures [60,61]. Another essential aspect to consider would be the possible caramelization of carbohydrates due to the effect of high temperatures, which would contribute to a more significant darkening of the products obtained [62,63].

### 3.5. Total Organic Carbon

**Figure 4:**
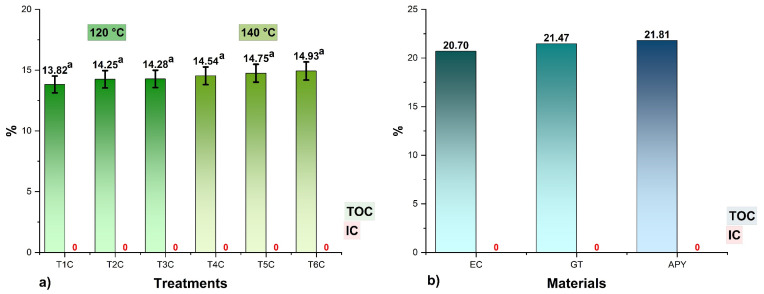
Total organic carbon (TOC) and total inorganic carbon (IC) content in, (**a**) the encapsulates at 120 °C (T1C, T2C, and T3C) and 140 °C (T4C, T5C, and T6C); (**b**) EC, spray-dried *C. porcellus* erythrocytes; GT, spray-dried tare gum; APY, starch of native potato variety yanapalta.

The TOC results are shown in Figure 4, where it could be seen that the values in the encapsulates varied between 13.82 and 14.93%, no significant differences were observed between the treatments for each input temperature (*p* > 0.05), and no amounts of inorganic carbon (IC) were reported because the samples were totally organic. It was also noted that the higher the proportions of encapsulants, the higher the TOC contents in the treatments since carbon atoms are a structural part of polypeptides, glucides, lipids, and fibers [44,57,64], so their determination would allow confirming the presence of these molecules in the spray-dried products [16,58,65,66].

### 3.6. SEM-EDX Analysis

**Figure 5:**
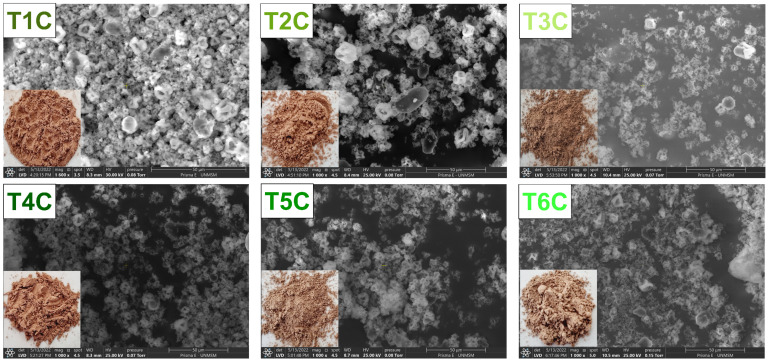
SEM images of the encapsulation at 120 °C (T1C, T2C, and T3C) and 140 °C (T4C, T5C, and T6C) at 1000× magnification.

Figure 5 shows the SEM images of the encapsulates and it could be seen irregular particles of different shapes and sizes. That is typical of the spray drying process [58,67,68,69,70,71,72], which presented larger particles and larger surface openings at an inlet air temperature of 140 °C [55,73], influenced by the feed characteristics and drying parameters [55,74]. Also, it could be due to the evaporation of the solvent during atomization [75], which would have produced a shrinkage and loss of sphericity in the particles [76]. The increase in temperature caused an increase in the drying rate and, therefore, the formation of holes in the particle [55,77]; on the contrary, lower temperatures would originate more consistent particles [78]. Nevertheless, the presence of amorphous structures in the encapsulates would be because of the use of native gum as a matrix [66,79].

**Figure 6:**
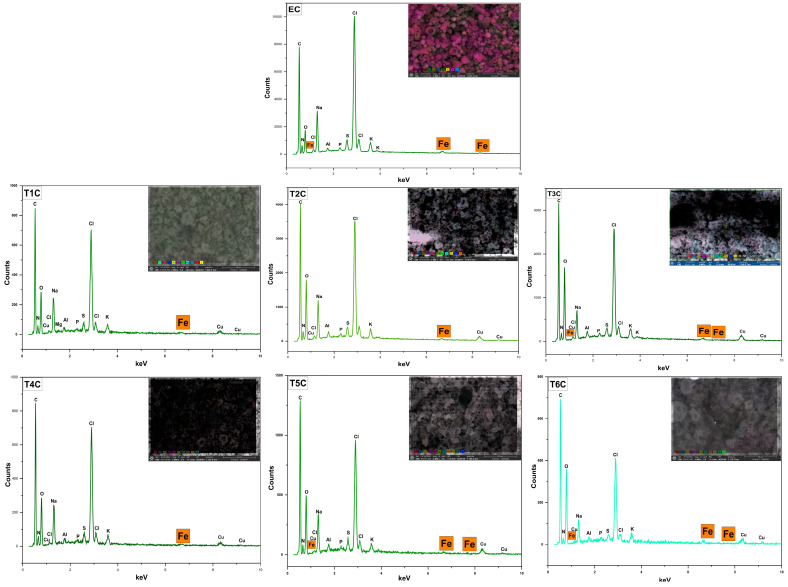
SEM-EDX analysis of erythrocytes (EC), encapsulated at 120 °C (T1C, T2C, and T3C) and 140 °C (T4C, T5C, and T6C).

Figure 6 shows the surface analysis of the atomized samples by SEM-EDX, which was conducted to confirm the presence of iron in the erythrocytes and encapsulates. It was observed an increase in the inlet temperature to 140 °C produced an increase in the surface amount of iron in the encapsulates. This increase in temperature caused a more significant size of the microparticles, which would allow the encapsulation of a greater amount of minerals inside and outside the atomized particles [55]. These results are related to the data obtained through the ICP OES in the present investigation.

### 3.7. Particle Size and ζ Potential

The size of the encapsulates and the ζ potential are observed in Table 5. The particle size determined by DLS varied between 541.6 and 903.7 nm for the NICOMP distribution and between 817.1 and 1672.2 nm for the Gaussian distribution. More than two peaks were also observed at T3C and T6C; and they were attributable to the spray drying that generates chemical and electrostatic interactions typical of the process, which would promote the agglomeration of particles of different sizes (including 39.8 and 122 nm), further influenced by the structural modification of the encapsulates, which were solubilized in water, giving rise to particles of different diameters from polypeptides, lipids and glucides [64,80]. The treatment with the highest proportion of encapsulants at 140 °C showed the largest particle size, while at 120 °C, the sizes were smaller [55,60,81].

The ζ potential is the surface charge obtained when particles are dissolved in a colloidal solution, whose stability depends on the values obtained and can vary between −100 and +100 mV. In the present study, values between −0.4 and −9.17 mV were obtained, which would indicate that the solutions formed are unstable with a tendency to coagulate and precipitate [82], probably due to the presence of different interactions, such as the case of hydrogen bridges, Van der Walls forces and hydrophobic bonds [31,83]. The ζ potential in the encapsulates was proportionally more negative with increasing inlet temperature and amounts of encapsulants. Also, the presence of guinea pig erythrocytes as the core would explain the appearance of anionic groups that would contribute to the total negative charge. In addition, it could also be since the ECs would have been trapped superficially in the particles, which would indicate their presence in a polymeric matrix [28,84]. The above is related to the data obtained through SEM-EDX, in which the presence of superficial iron was confirmed due to the concavities in the particles.

### 3.8. FTIR Analysis

**Figure 7:**
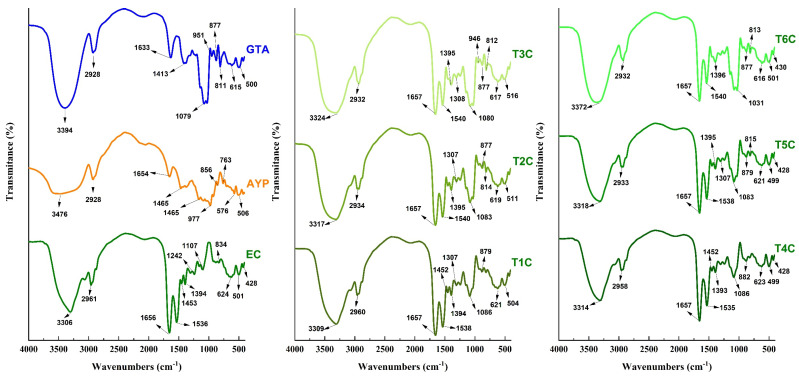
Infrared spectra in tara gum (GTA), native potato starch of the yanapalta variety (AYP), erythrocytes (EC) and encapsulated at 120 °C (T1C, T2C, and T3C) and 140 °C (T4C, T5C, and T6C).

It was carried out to confirm that the cores were successfully encapsulated in the matrices [31], using an approved methodology for reading and interpreting the infrared spectra [85]. In Figure 7, the typical spectra of the analyzed materials are appreciated; in the case of GTA, APY, and EC, strong bands of 3394, 3476, and 3308 cm^−1^ were appreciated, which would correspond to the OH hydroxyl group given by stretching vibration [79,86], wave numbers of similar intensity were observed in all the encapsulations and varied between 3309 and 3372 cm^−1^. On the other hand, an intense vibrational stretching band at 1079 cm^−1^ was also observed in GTA, which would correspond to the C-O bond of the carboxylic acid. This band is also present in all encapsulates between 1031 and 1086 cm^−1^ [79].

The encapsulated samples also observed some spectral bands presented in the erythrocytes. The wave number of 2961 cm^−1^ of the erythrocytes also appeared in the other treatments between 2932 and 2960 cm^−1^, corresponding to characteristic C-H stretches in atomized erythrocytes from animal blood and encapsulated in polymeric matrices. The presence of spectral bands at 1536 and 1656 cm^−1^ observed in the erythrocytes were also present in the encapsulated particles between 1535–1540 cm^−1^ and at 1657 cm^−1^; which would confirm the presence of the amide group I bond by C-O vibrational stretching and the N-H bond by bending vibration of the amide group II [28,31,39]. The bands around 615 cm^−1^ could be attributable to the pyranose ring of tara gum which is present in all the encapsulates [79,87,88]. Those mentioned above would ensure that the encapsulation was successful since the results were quite similar to those reported by other authors [28,31]. It was also noted that the increase from 120 to 140 °C in the air inlet temperature produced a modification in the intensities of the functional groups [55].

### 3.9. Thermal Analysis

**Figure 8:**
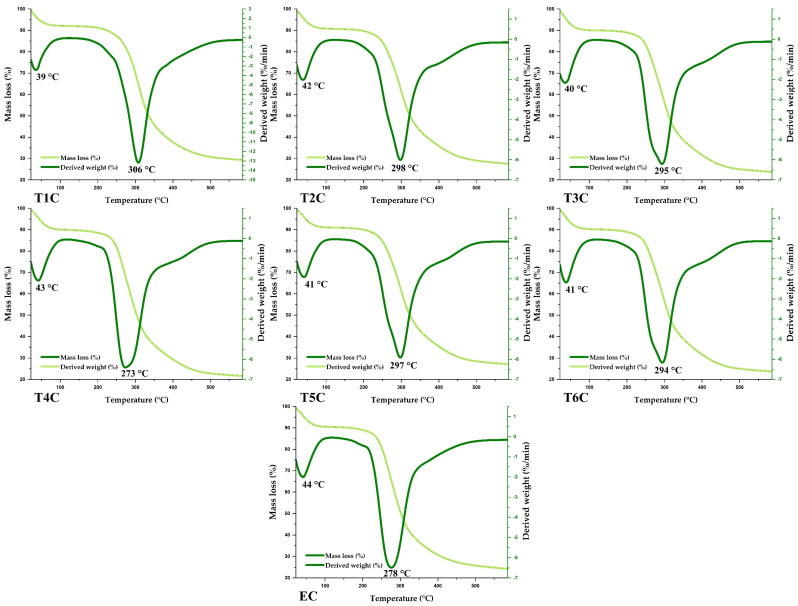
TG and DTA of encapsulated at 120 °C (T1C, T2C, and T3C) and 140 °C (T4C, T5C, and T6C), and erythrocytes (EC).

The encapsulates and erythrocytes had similar thermal behavior (Figure 8). According to the TGA and DTG curves, similar mass losses were observed in the same temperature range. It was also observed that the first event occurred between 39 and 44 °C, with a weight loss of about 4%, mainly due to the elimination of water and various volatile compounds. On the other hand, a second event was observed between 273 and 306 °C, with a mass loss of about 25 and 42%, caused mainly by the decomposition of organic compounds such as proteins, lipids, and carbohydrates; at higher temperatures, the other components of the encapsulated matrix would be lost [89,90,91].

### 3.10. In Vitro Bioavailability

**Figure 9:**
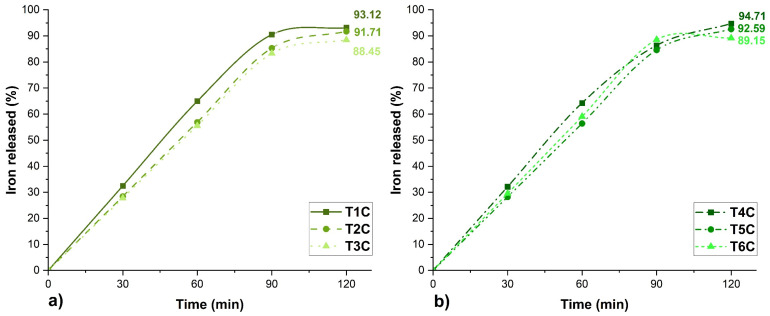
Erythrocyte encapsulations, (**a**) encapsulated at 120 °C and (**b**) encapsulated at 140 °C.

Figure 9 shows the in-vitro iron release profile of the encapsulates. At 120 min, the T4C treatment presented the highest result of 94.71%, followed by the T1C treatment, whose value was 91.71%. Likewise, high values of iron release could also be appreciated in the other treatments (T2C, T3C, T5C, and T6C), whose bioavailability in the small intestine is essential to reduce the risk of suffering from anemia since only 1 to 2 mg of iron is absorbed in this part of the human body [15]. 

Considering iron release in-vitro, the treatment T4C at 140 °C would be the best. The results obtained in the present investigation were similar to those reported for iron encapsulations in potato starch-maltodextrin matrices [92], chitosan-eudragit [93], Eudraguard [94], and dextrin [95]. However, more in-depth studies of iron bioavailability are recommended, considering its release at the gastric and intestinal level [28,32], including complex studies of gastrointestinal behavior [96]. 

**Figure 10:**
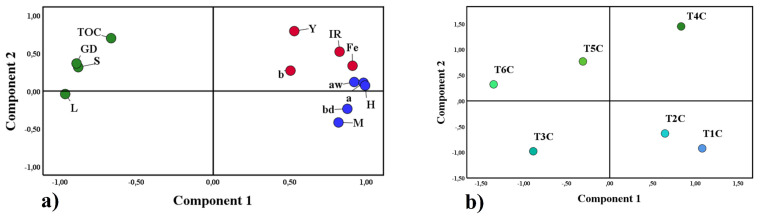
Principal component analysis, properties studied (**a**), and treatments (**b**).

Principal component analysis (PCA) is a statistical method that allows the reduction of many study variables and visualizes the close relationship between complex factors [97], for this reason, it was applied in this research. Figure 10a shows the first group in blue, formed by water activity (Aw), moisture (M), hygroscopicity (H), bulk density (bd), and chroma a; on the other hand, the second group in red was formed by the affinity between iron content (Fe), yield (Y), iron release (IR) and chroma b; both groups were associated with component 1. Finally, the third group in green was formed by total organic carbon (TOC), Gaussian particle size distribution (GD), solubility (S), and lightness (L) associated with component 2. Those mentioned above would confirm the close relationship between the variables of each group, which influenced the results obtained in the encapsulates. On the other hand, Figure 10b corroborates the significant difference between the treatments studied.

## 4. Conclusions

A high concentration of heme iron from guinea pig blood erythrocytes (20% *w/v*) was encapsulated using a mixture of tara gum and native potato starch (5, 10, and 20% *w/v*) as a coating material; the process was performed by spray drying in an aqueous solution at 120 and 140 °C. As a result, high iron contents were obtained in the erythrocytes and encapsulated; also, a high rate of bioavailability in vitro for the T4C treatment was found. Suitable physicochemical and structural properties were also obtained, observing that the particle sizes were found at the nanometric level, with a tendency to agglomerate and precipitate in colloidal solutions. SEM-EDX confirmed the presence of surface iron. FTIR analysis was used to analyze the entrapment of the core in the polymeric matrix, which was confirmed because many functional groups of the materials were also observed in the encapsulates. Finally, the present research showed that the inlet temperature and the amount of encapsulant had an effect on the properties studied, and the novelty of the combination of the matrices studied was also analyzed. Therefore, the results would allow the use of economical raw materials for inclusion as ingredients in the fortification of food products for the fight against iron deficiency anemia, affecting many developing countries. One limitation of the study was the non-inclusion of encapsulates in food products due to the economic and food safety studies that still need to be developed in detail.

## Figures and Tables

**Table 1:** Erythrocyte encapsulation treatments.

Treatments	Temperature	SST	EC	APY	GTA
	°C	%	%	%	%
T1C	120	25	20	4	1
T2C		30	20	8	2
T3C		40	20	16	4
T4C	140	25	20	4	1
T5C		30	20	8	2
T6C		40	20	16	4

Where: SST, total soluble solids; EC, spray-dried *C. porcellus* erythrocytes; APY, starch of native potato variety yanapalta and GTA, spray-dried tare gum.

**Table 2:** Moisture, Aw, and bulk density of erythrocytes and encapsulates.

	Moisture (%)				Aw				Bulk Density			
	$\bar{x}$	±	SD	CV	$\bar{x}$	±	SD	CV	$\bar{x}$	±	SD	CV
EC	11.30	±	0.44	3.85	0.43	±	0.002	0.35	0.23	±	0.007	2.92
T1C	8.61	±	0.09 ^a^	1.03	0.40	±	0.003 ^a^	0.63	0.24	±	0.001 ^a^	0.30
T2C	6.86	±	0.07 ^b^	0.96	0.41	±	0.003 ^b^	0.62	0.24	±	0.002 ^a^	1.13
T3C	4.85	±	0.12 ^c^	2.43	0.35	±	0.001 ^c^	0.33	0.22	±	0.001 ^b^	0.41
T4C	5.69	±	0.05 ^a^	0.96	0.40	±	0.001 ^a^	0.29	0.24	±	0.003 ^a^	1.09
T5C	5.52	±	0.18 ^a^	3.31	0.38	±	0.002 ^b^	0.40	0.20	±	0.003 ^b^	1.29
T6C	4.73	±	0.11 ^b^	2.32	0.36	±	0.001 ^c^	0.16	0.17	±	0.005 ^c^	3.04

Where: EC, spray-dried *C. porcellus* erythrocytes; T1C, T2C y T3C encapsulated at 120 °C, and T4C, T5C y TC6 encapsulated at 140 °C; $\bar{x}$, arithmetic mean; SD, standard deviation; CV, coefficient of variability. Different letters indicate a significant difference.

**Table 3:** Solubility and hygroscopicity of erythrocytes and encapsulates.

	Solubility (%)				Hygroscopicity (%)			
	$\bar{x}$	±	SD	CV	$\bar{x}$	±	SD	CV
EC	72.08	±	0.47	0.65	36.66	±	0.59	1.60
T1C	43.22	±	0.83 ^a^	1.92	32.94	±	1.20 ^a^	3.65
T2C	44.27	±	2.76 ^a^	6.23	30.13	±	1.39 ^b^	4.62
T3C	49.74	±	2.54 ^b^	5.11	22.56	±	1.26 ^c^	5.59
T4C	45.11	±	1.06 ^a^	2.36	31.95	±	0.60 ^a^	1.88
T5C	55.21	±	4.84 ^b^	8.77	26.03	±	1.48 ^b^	5.71
T6C	57.18	±	1.20 ^b^	2.10	22.05	±	0.86 ^c^	3.91

Where: EC, spray-dried *C. porcellus* erythrocytes; T1C, T2C y T3C encapsulated at 120 °C, and T4C, T5C y TC6 encapsulated at 140 °C; $\bar{x}$, arithmetic mean; SD, standard deviation; CV, coefficient of variability. Different letters indicate a significant difference.

**Table 4:** Yield and color of tara gum, native potato starch, erythrocytes and encapsulates.

	Yield				Color											
	(%)				*L*				a				b			
	$\bar{x}$	±	SD	CV	$\bar{x}$	±	SD	CV	$\bar{x}$	±	SD	CV	$\bar{x}$	±	SD	CV
GT	12.31	±	0.64	5.22	91.02	±	0.06	0.07	0.06	±	0.01	10.19	3.52	±	0.05	1.34
APY	12.34	±	0.22	1.78	92.69	±	0.07	0.07	−0.16	±	0.01	−6.25	1.66	±	0.03	1.51
EC	29.18	±	1.19	4.06	39.37	±	0.26	0.65	15.18	±	0.08	0.49	21.45	±	0.08	0.36
T1C	52.73	±	0.79 ^a^	1.49	54.17	±	0.12 ^a^	0.23	9.82	±	0.06 ^a^	0.56	18.99	±	0.06 ^a^	0.29
T2C	49.32	±	0.93 ^b^	1.88	53.88	±	0.16 ^a^	0.29	9.61	±	0.07 ^b^	0.75	19.68	±	0.01 ^b^	0.03
T3C	47.84	±	1.39 ^b^	2.92	59.38	±	0.18 ^b^	0.30	7.20	±	0.10 ^c^	1.34	19.12	±	0.06 ^c^	0.30
T4C	58.73	±	1.30 ^a^	2.21	54.37	±	0.06 ^a^	0.10	9.90	±	0.02 ^a^	0.23	20.22	±	0.09 ^a^	0.46
T5C	53.29	±	1.11 ^a^	2.09	56.40	±	0.06 ^b^	0.11	8.61	±	0.05 ^b^	0.52	18.50	±	0.01 ^b^	0.06
T6C	50.33	±	3.80 ^a^	7.55	61.38	±	0.02 ^c^	0.03	6.38	±	0.02 ^c^	0.33	18.96	±	0.03 ^c^	0.18

Where: GT, spray-dried tare gum; APY, starch of native potato variety yanapalta; EC, spray-dried *C. porcellus* erythrocytes; T1C, T2C y T3C encapsulated at 120 °C, and T4C, T5C y TC6 encapsulated at 140 °C; $\bar{x}$, arithmetic mean; SD, standard deviation; CV, coefficient of variability. Different letters indicate a significant difference.

**Table 5:** Particle size and ζ potential.

Treatments	NICOMP Distribution			Gaussian Distribution			ζ Potential (mV)
	Peak	Size (nm)	%	$\bar{x}$	SD	CV	
T1C	1	541.6	100.0	817.1	67.98	8.32	−0.40
T2C	1	677.0	100.0	1076.8	82.05	7.62	−0.83
T3C	1	122.0	6.1	1595.5	115.67	7.25	−1.79
	2	188.3	9.9				
	3	902.5	84.0				
T4C	1	900.2	100.0	1331.8	121.64	8.26	−2.19
T5C	1	902.8	100.0	1472.7	104.81	7.87	−8.60
T6C	1	39.8	1.2	1672.2	132.27	7.91	−9.17
	2	199.3	23.0				
	3	903.7	75.8				

Where: T1C, T2C, and T3C encapsulated at 120 °C and T4C, T5C and TC6 encapsulated at 140 °C; $\bar{x}$, arithmetic mean; SD, standard deviation; CV, coefficient of variability.

## Data Availability

The data presented in this study are available in this same article.
